# P04.16. Integrative medicine practice patterns across the US: results from a survey of 29 centers

**DOI:** 10.1186/1472-6882-12-S1-P286

**Published:** 2012-06-12

**Authors:** D Abrams, B Horrigan, S Lewis, M Acree, C Pechura

**Affiliations:** 1 University of California, San Francisco, San Francisco, USA; 2 Bravewell Collaborative, Minneapolis, USA

## Purpose

Integrative medicine is emerging as a vital part of the US healthcare system. To better understand how integrative medicine is actually being practiced, a study was undertaken to: (1) Describe the patient populations and health conditions most commonly treated; (2) Investigate the interventions utilized; and (3) Ascertain the distinctiveness of treatments used for different conditions; (4) Assess concordance between centers in treating a given condition.

## Methods

All 29 integrative medicine centers approached to participate completed a REDCap-based survey in summer 2011. Survey data was exported into SAS for analysis. To assess the degree to which different treatments were used for different conditions, the frequencies of treatments for each condition were ranked. Then, for each pair of conditions, the correlation between treatment ranks was calculated.

## Results

All of the centers are affiliated with a hospital/ health care system or medical school. Twenty-six of the 29 centers offer consultative care. Thirteen offer primary care. All centers provide services to adults with 28 offering geriatric services, 25 adolescent, 21 OB-GYN and 18 pediatrics. Overall 63% of patients seen are self-referred. From a list of 20 clinical conditions, respondents chose the top five which they have the greatest success treating as: chronic pain (75%), gastrointestinal conditions (59%), depression/anxiety (55%), stress (52%) and cancer (52%). Of 34 integrative interventions listed in the survey, those utilized most frequently across the 20 conditions include (in descending order): food/nutrition, supplements, yoga, meditation, TCM/acupuncture, massage and pharmaceuticals. Treatments were consistent for like and distinct for different conditions (e.g., correlation between allergies and asthma .92, between allergies and acute pain .36). Concordance between the individual centers in treating a given condition was significant.

## Conclusion

Integrative medicine is practiced in diverse sites across the country with high levels of concordance of interventions for specific conditions suggesting that practice is driven by an evidence base.

